# A fatal masquerade in pneumonia: Ruptured thoracic aortic aneurysm

**DOI:** 10.1002/ccr3.5285

**Published:** 2022-01-11

**Authors:** Ching‐Han Liu, Shih‐Chung Huang, Ching‐Tsai Hsu

**Affiliations:** ^1^ Division of Cardiology Department of Medicine Kaohsiung Armed Forces General Hospital Kaohsiung Taiwan; ^2^ Division of Cardiology Department of Medicine National Defense Medical Center Tri‐Service General Hospital Taipei Taiwan; ^3^ Division of Cardiology Department of Medicine Taoyuan Armed Forces General Hospital Taoyuan Taiwan

**Keywords:** endovascular aortic repair, hemoptysis, pneumonia, ruptured thoracic aortic aneurysm

## Abstract

We described an 87‐year‐old man who presented with fever and hemoptysis. Nosocomial pneumonia was initially suspected. However, the patient had worsening hemoptysis despite defervescence. Chest computed tomography disclosed ruptured thoracic aortic aneurysm. Emergent surgery was then commenced for adequate treatment.

## Graphical abstract

An 87‐year‐old man presented with fever, hemoptysis, and left upper lung infiltrate on chest radiograph. Nosocomial pneumonia was initially suspected. Chest computed tomography performed hours later for excluding an intrathoracic pathology revealed ruptured thoracic aneurysm. Prompt surgical intervention was successfully performed to treat this fatal condition.

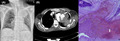

## 1 CASE DESCRIPTION

**FIGURE 1:**
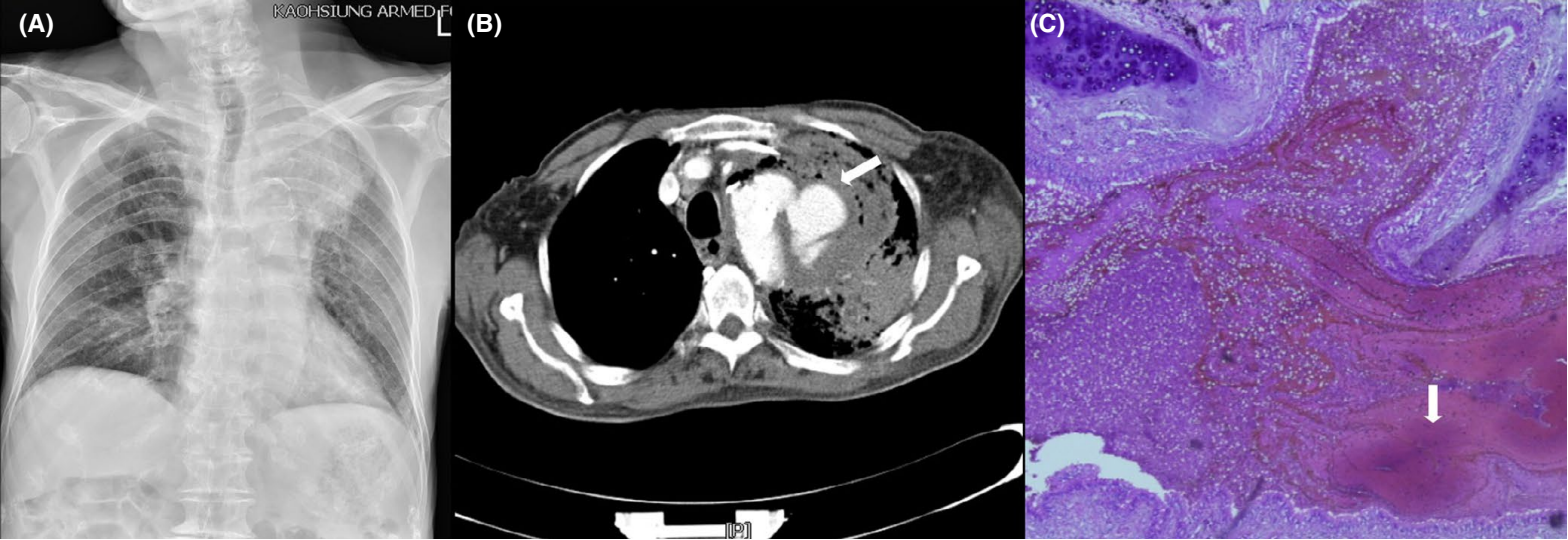
(A) Chest radiograph of an 87‐year‐old man obtained 3 h after presentation to the emergency department for survey of worsening hemoptysis. Evident progression of opacification over left upper lung was noted. (B) Computed tomography of chest was promptly performed, which demonstrated one ruptured thoracic aortic aneurysm (arrow). (C) Surgical pathology demonstrated whole‐layer inflammation of aortic wall and hemorrhage into lung parenchyma, causing hemobronchus and pulmonary hemorrhage (arrow)

An 87‐year‐old man presented to the emergency department with productive cough and hemoptysis. He had been hospitalized 1 month ago for urinary tract infection. He had mild fever of 37.8°C with mild respiratory embarrassment. Chest radiograph demonstrated opacification in the left upper lung. Due to the history of recent hospitalization, nosocomial pneumonia was suspected with empiric antimicrobial therapy commenced. Despite defervescence, his hemoptysis worsened. Progression of left upper lung consolidation was noted 3 h later (Figure 1A). Chest computed tomography (CT) demonstrated extravasation of contrast over aortic arch, which was suggestive of a ruptured aortic aneurysm (Figure 1B). Emergent hybrid surgery comprising of total arch replacement with thoracic endovascular aortic repair and wedge resection of the left upper lung was performed. Pathologic examination of the aorta and lung tissues demonstrated transmural inflammation of thoracic aorta, hemobronchus, and pulmonary hemorrhage (Figure 1C).

Ruptured thoracic aortic aneurysm carries a high mortality rate of 50%–80% even with surgical intervention.^1^ Hemoptysis is a rare presenting symptom.^2^ The outcome can worsen if correct diagnosis is delayed. This underscores the importance of performing CT as a first‐line investigation in case of atypical presentation of pneumonia to rule out serious pathologies.

## CONFLICT OF INTEREST

None declared.

## AUTHOR CONTRIBUTIONS

CH Liu conceived the idea and wrote the manuscript; SC Huang edited the manuscript; CT Hsu collected the clinical data.

## CONSENT

An informed consent for publication was obtained from the patient and his family. Written informed consent was obtained from the patient to publish this report in accordance with the journal's patient consent policy.

## Data Availability

Data available upon request.
